# Data on inheritance of race 2 anthracnose resistance in watermelon *(Citrullus spp.)* biparental mapping populations

**DOI:** 10.1016/j.dib.2022.108546

**Published:** 2022-08-19

**Authors:** Bed Prakash Bhatta, Edgar Correa, Takshay Patel, Todd C. Wehner, Kevin M. Crosby, Michael J. Thomson, Subas Malla

**Affiliations:** a Department of Horticultural Sciences, Texas A&M University, College Station, TX 77843, USA; b Texas A&M AgriLife Research and Extension Center, 1619 Garner Field Road, Uvalde, TX, 78801, USA; c Department of Horticultural Science, North Carolina State University, Raleigh, NC 27695, USA; d Department of Soil and Crop Sciences, Texas A&M University, College Station, TX 77843, USA

**Keywords:** Race 2 anthracnose, Watermelon, Genetic studies, Mode of inheritance

## Abstract

Anthracnose of watermelon is caused by a fungal pathogen *Colletotrichum orbiculare*. We generated F_2_ individuals from three different populations: Population 1 (PI 189225 x ‘New Hampshire Midget’), Population 2 (‘Perola’ x PI 189225), and Population 3 (‘Verona’ x PI 189225). The biparental F_2_ populations, parents and F_1_ individuals were inoculated with an isolate of race 2 anthracnose isolated from watermelon. Leaf lesions were visually rated seven days post inoculation on a scale of 0% (no lesion) to 100% (dead true leaf). Here we present the datasets obtained after the disease inoculation. The distribution of data obtained was visualized using histograms and goodness-of-fit was tested using Chi-Square. These datasets provide information on the mode of inheritance of race 2 anthracnose resistance in watermelon.

**Specifications Table**

**Table:** 

Subject	Biological Sciences
Specific subject area	Genetics; Plant Science
Type of data	Table, Figure
How the data were acquired	Visual rating on a scale of 0 to 100% after seven days of inoculation of watermelon leaves with race 2 anthracnose pathogen
Data format	Raw Analyzed
Description of data collection	Three biparental mapping populations were generated. Population 1 had 190 F_2_ individuals and 16 F_1_ individuals. Population 2 and Population 3 had 89 and 101 F_2_ individuals, respectively. A race 2 anthracnose isolate was used at a concentration of 100,000 spores/mL to inoculate three-week old watermelon seedlings. Disease rating (percent leaf lesion) was done visually seven days post inoculation.
Data source location	Institution: Texas A&M AgriLife Research and Extension Center City/Town/Region: Uvalde, Texas Country: USA Latitude and longitude (and GPS coordinates, if possible) for collected samples/data: 29.2097° N, 99.7862° W
Data accessibility	Disease rating data for Population 1, Population 2, and Population 3 have been stored at Mendeley Data repository under the folder ‘**Disease rating_Population123**’. The folder contains files: **Pop1_PI 189225 X NHM.xlsx; Pop2_Perola X PI 189225.xlsx; Pop3_Verona X PI 189225.xlsx**, respectively. Histograms of the disease rating data (Fig.1 in the article) has also been shared separately at Mendeley Data repository for Population 1, Population 2, and Population 3 in the folder ‘**Histograms_Population123**’. The folder contains files **Fig1a_Pop1_F2.tiff; Fig1b_Pop2_F2.tiff; Fig1c_Pop3_F2.tiff**, respectively. Repository name: Mendeley Data Data identification number: 10.17632/jp6frx2gwf.2 10.17632/sj6c52bb36.1 Direct URL to data: https://data.mendeley.com/datasets/jp6frx2gwf https://data.mendeley.com/datasets/sj6c52bb36

**Value of the Data**

• These disease rating data of three watermelon biparental mapping populations provide information on the mode of inheritance for race 2 anthracnose resistance in watermelon.

**Fig. 1:**
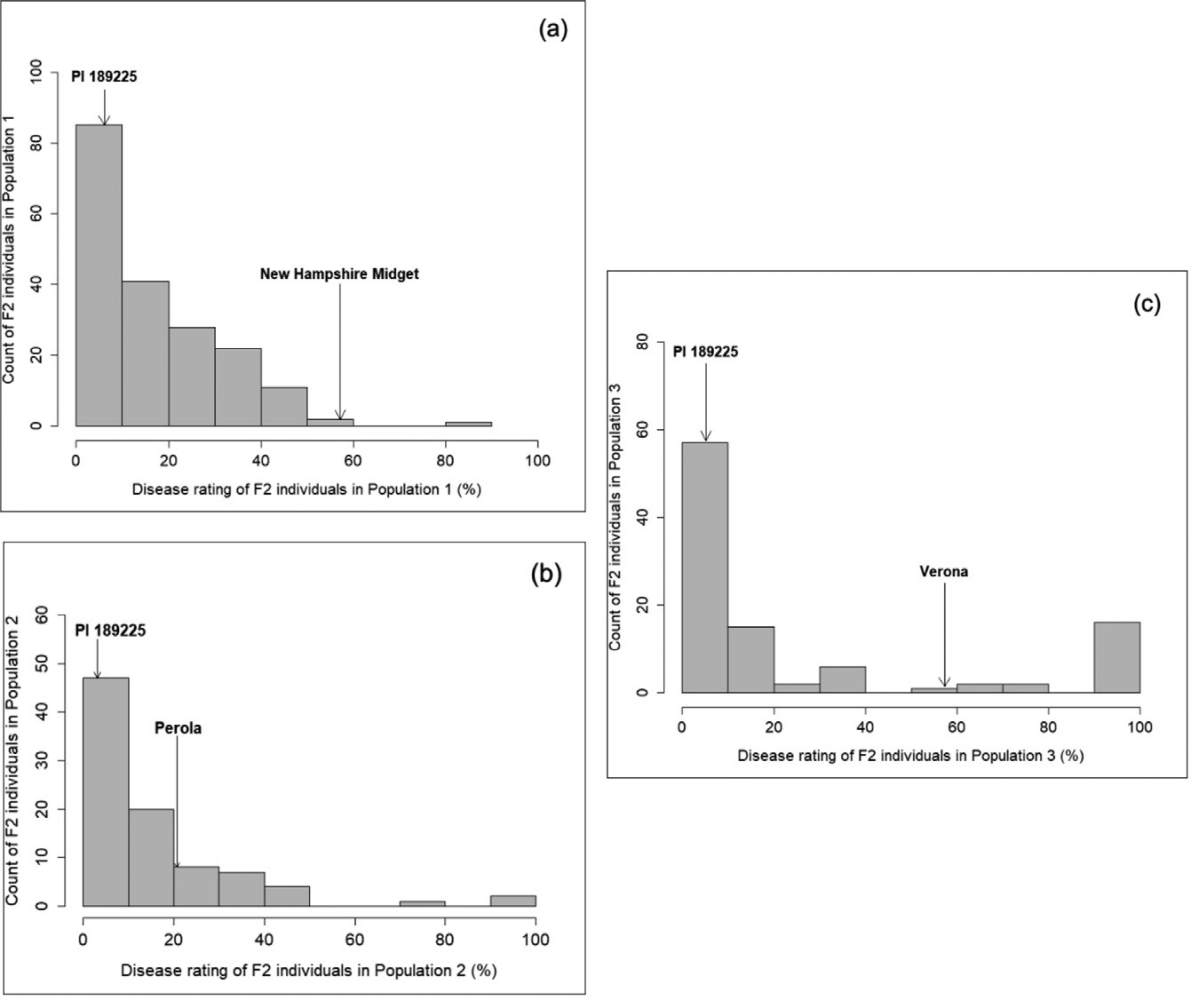
Histograms showing distribution of disease rating in F_2_ individuals of three watermelon biparental mapping populations: *(a)* Population 1, *(b)* Population 2, and *(c)* Population 3 inoculated with race 2 anthracnose. These files are available as **Fig1a_Pop1_F2.tiff, Fig1b_Pop2_F2.tiff**, and **Fig1c_Pop3_F2.tiff** in the Mendeley data repository.
• These datasets may be useful to researchers working in cucurbit breeding/genetics and aiming to understand genetics behind race 2 anthracnose resistance.
• These datasets may be used to compare mode of inheritance of race 2 anthracnose resistance in watermelon with populations involving different parents.
• These phenotypic disease rating datasets may be combined with their genotypic data to find quantitative trait loci (QTL) associated with race 2 anthracnose resistance in watermelon.
• Information from these datasets may contribute to marker assisted selection (MAS) in watermelon for race 2 anthracnose resistance.

## 1 Data Description

Here we report the disease rating (percent leaf lesion) data obtained after inoculating three watermelon biparental mapping populations with race 2 anthracnose (files shared at Mendeley Data repository: **Pop1_PI 189225 X NHM.xlsx; Pop2_Perola X PI 189225.xlsx; Pop3_Verona X PI 189225.xlsx**) [1].

‘**Pop1_PI 189225 X NHM.xlsx**’ contains disease rating data for Parent 1 (PI 189225), Parent 2 (New Hampshire Midget), F_1_ and F_2_ individuals. ‘**Pop2_Perola X PI 189225.xlsx**’ contains disease rating data for Parent 1 (Perola), Parent 2 (PI 189225), and F_2_ individuals. ‘**Pop3_Verona X PI 189225.xlsx**’ contains disease rating data for Parent 1 (Verona), Parent 2 (PI 189225), and F_2_ individuals. The distribution of phenotypic data obtained for F_2_ individuals in each population were visualized using histograms and goodness-of-fit was tested using Chi-square test. For all the three populations, the histograms indicated a non-normal distribution (Fig. 1a, b, and c). X-axis in the histogram shows disease rating (percent leaf lesion) and Y-axis shows count of the F_2_ individuals. The histograms also show average disease rating of the parents in each population using the drop-down arrows.

The cutoff for resistance and susceptibility in the disease rating data of F_2_ individuals was determined by adding 10 units to the highest rating of the resistant parent (PI 189225) in each population. Based on this criterion, the cutoffs for Population 1, 2, and 3 were 30, 25, and 35 respectively. Using these cutoffs, the count of resistant (*R*): susceptible (*S*) individuals for Population 1, 2, and 3 was 140*R*:50*S*, 67*R*:22*S*, and 74*R*:27*S*, respectively. The F_2_ individuals in Population 1 (χ ^2^_3:1_ = 0.17, *P* = 0.67), Population 2 (χ^2^_3:1_ = 0.0037, *P* = 0.95), and Population 3 (χ^2^_3:1_ = 0.16, *P* = 0.68) failed to reject the null hypothesis of 3:1 (*R:S*) segregation ratio. *P*-value ≥ 0.05 indicates there is no significant difference between observed versus expected Mendelian ratios.

## 2 Experimental Design, Materials and Methods

### 2.1 Plant material

F_2_ individuals were generated by selfing F_1_ individuals obtained from the crosses of Population 1 (PI 189225 x ‘New Hampshire Midget’), Population 2 (‘Perola’ x PI 189225), and Population 3 (‘Verona’ x PI 189225). PI 189225 is resistant to race 2 anthracnose [2,3]. Seeds for Population 1 (parents, F_1_ and F_2_) were obtained from Dr. Todd Wehner (North Carolina State University). Population 2 and 3 were developed at Texas A&M AgriLife Research and Extension Center, Uvalde, TX, USA.

### 2.2 Inoculum preparation and inoculation

The inoculations were conducted during spring and summer of 2019 in Uvalde, TX. The race 2 anthracnose isolate *WmColl4*, kindly provided by Dr. A. Keinath (Clemson University) was used to inoculate the three-week old watermelon seedlings. The isolate was grown on half-strength dextrose agar for 7 to 14 days, harvested with distilled water, and poured into a beaker through a cheese cloth. The watermelon seedlings were inoculated with a 100,000 spores/mL concentration of the fungus in a greenhouse using a CO_2_ sprayer at 30 PSI. Inoculated seedlings were moved into a humidity chamber (approximately 100% relative humidity, temperature: 22 to 24°C) for 48 hours and later placed under natural light at greenhouse (day temperature: 30°C and night temperature: 25°C).

### 2.3 Disease rating

**Fig. 2:**
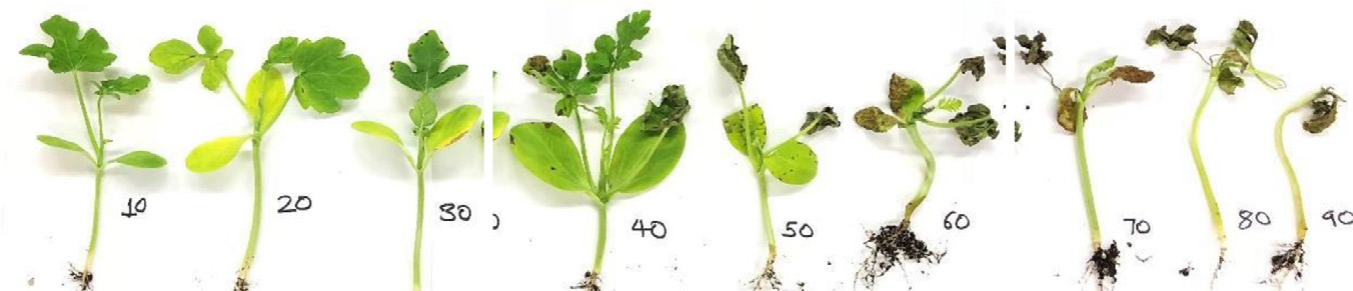
Anthracnose disease severity on watermelon seedling rated on a scale of 0 to 100% with an interval of 10% (Patel, 2019) [4].

The biparental F_2_ populations were rated at seven days post inoculation following the protocol of Patel, 2019 [4,5]. True leaves from each watermelon seedling were rated for percent leaf lesion on a scale of 0 to 100% at an interval of 10, where 0% indicating no lesion and 100% indicating complete necrosis of true leaves (Fig. 2).

### 2.4 Data visualization and analysis

Histograms were generated for the disease rating data of F_2_ individuals in three biparental mapping populations using ‘hist’ function and goodness-of-fit was tested using ‘chisq.test’ functions in RStudio (2021.09.0 Build 351).

## CRediT authorship contribution statement

**Bed Prakash Bhatta:** Methodology, Software, Data curation, Investigation. **Edgar Correa:** Methodology, Data curation, Investigation. **Takshay Patel:** Conceptualization, Methodology, Writing – review & editing. **Todd C. Wehner:** Conceptualization, Supervision, Validation, Writing – review & editing, Funding acquisition. **Kevin M. Crosby:** Writing – review & editing. **Michael J. Thomson:** Writing – review & editing. **Subas Malla:** Conceptualization, Data curation, Investigation, Supervision, Validation, Writing – review & editing, Funding acquisition.

## Declaration of Competing Interest

The authors declare that they have no known competing financial interests or personal relationships that could have appeared to influence the work reported in this paper.

## Data Availability

Histograms_Population123 (Original data) (Mendeley Data). Disease rating_Population123 (Original data) (Mendeley Data).
